# Experimental Measurement and Modeling of Hg(II) Removal from Aqueous Solutions Using *Eucalyptus globulus* Bark: Effect of pH, Salinity and Biosorbent Dosage

**DOI:** 10.3390/ijms20235973

**Published:** 2019-11-27

**Authors:** Elaine Fabre, Carlos Vale, Eduarda Pereira, Carlos M. Silva

**Affiliations:** 1 CICECO (Aveiro Institute of Materials) and CESAM (Centro de Estudos do Ambiente e do Mar), University of Aveiro, 3810-193 Aveiro, Portugal; elainefabre@ua.pt; 2 CIIMAR (Interdisciplinary Centre of Marine and Environmental Research), University of Porto, 4050-123 Matosinhos, Portugal; carlos.vale@ciimar.up.pt; 3 CESAM and LAQV (Laboratório associado para a Química Verde)-REQUIMTE (Rede de Química e Tecnologia), University of Aveiro, 3810-193 Aveiro, Portugal; 4 CICECO, University of Aveiro, 3810-193 Aveiro, Portugal

**Keywords:** *Eucalyptus globulus* bark, mercury, modelling, response surface methodology, sorption

## Abstract

Different experimental conditions were tested in order to optimize the Hg(II) removal by *Eucalyptus globulus* bark. Response surface methodology was applied to extract information about the significance of the factors and to obtain a model describing the sorption. The results were generated through the design of experiments by applying the methodology of a three-factor and three-level Box–Behnken design. The factors tested were pH (4.0, 6.5, and 9.0), salinity (0, 15, and 30), and biosorbent dosage (0.2, 0.5, and 0.8 g dm^−3^) to evaluate the Hg(II) removal using realistic conditions, such as contaminated natural waters with an initial Hg(II) concentration of 50 µg dm^−3^. The optimum response provided by the model was 81% of the metal removal under the optimal operating conditions: a pH value of 6.0, no salinity, and a biosorbent dosage of 0.55 g dm^−3^. Concerning the kinetic, the pseudo-second-order equation fitted better to the experimental results with $R^{2}$ between 0.973 and 0.996. This work highlights the promising valorization of this biomass, which is an industrial byproduct and makes available information about the influence of the variables for Hg(II) removal in water treatment processes.

## 1. Introduction

Mercury is a nondegradable toxic metal classified by the Agency for Toxic Substances and Disease Registry (ATSDR) as the third most dangerous substance. This list is elaborated, considering facts such as the toxicity, occurrence in the environment, and risks for human health [1]. Furthermore, under Directive 2013/39/EU of the European Parliament, mercury and its compounds are classified as priority substances, and their emissions must be progressively reduced and eliminated by 2021 [2]. This directive also encourages the development of innovative cheaper technologies for the improvement of water quality [2].

Major anthropogenic sources of mercury are effluents from chloralkali, pulp and paper, petroleum refining, and electric batteries and lamp production [3]. Technologies, such as membrane processes, chemical precipitation, flotation, coagulation–flocculation, and electrochemical techniques reduce metal content present in waters in a range of concentrations of mg dm^−3^ [4,5]. However, these conventional methods may be inadequate, expensive and generate secondary sludge, and most of the times are not effective to reach final low levels [6,7]. Sorption processes like adsorption and ion exchange are the most applied ones in industries, and despite their efficiency, the cost of a sorbent is a restraining factor for the implementation of this cleanup operation.

The biosorbents have been recognized as good options for tracing metal removal from waters. They are usually composed of cellulose, hemicellulose, and lignin, which have a high content of hydroxyl and carboxyl groups [8]. Due to abundant binding groups, their capacities can be equal or even greater compared with those of the conventional sorbents, which makes these materials promising sources for decontaminating toxic metals from wastewaters [8,9,10]. Balderas-Hernández et al. used 10 g dm^−3^ of *Allium cepa* L. for mercury removal and obtained a Hg(II) elimination of 99.4% from the solutions with 20 mg dm^−3^ of this metal [11]. Aman et al. studied the performance of rose flowers (*Rosa indica*) for mercury sorption, and they found a biosorbent uptake capacity of 11.91 mg g^−1^ [12]. *Phragmites australis* (dose of 20 g dm^−3^) was applied in sorption and removed 80% of Hg(II) from the solutions spiked with 10 mg of Hg(II) dm^−3^ [13]. Despite several works reported using biosorbents, only a few of them consider realistic low initial concentrations of mercury. These vestigial concentrations are most commonly used in aquatic bodies, and therefore, they are the conditions that must be pursued [14].

*Eucalyptus* is the most important source of biomass for paper pulp industries, which generate large amounts of biomass bark wastes. This byproduct has been used as a biosorbent for diverse metals uptake. *Eucalyptus globulus* (*E.* *globulus*) bark pretreated with sulfuric acid was successfully used for Pb(II) and Cd(II) removal, achieving capacities of 26.12 mg g^−1^ for Pb(II) and 35.65 mg g^−1^ for Cd(II) [15]. In another study, *E. camaldulensis Dehn*. bark was investigated for Cu(II) and Pb(II) sorption after impregnation with phosphoric acid and carbonization. The capacities found were 54.02 mg g^−1^ and 184.41 mg g^−1^ for Cu(II) and Pb(II), respectively [16]. Cr(VI) was completely removed by *E. globulus* bark biochar at a dose of 2 g dm^−3^ from contaminated groundwater (initial Cr(VI) concentration of 25 g dm^−3^) in the work of Choudhary et al. [17].

It is well documented that sorption performance is highly dependent on various operating conditions, such as pH, temperature, ionic strength, sorbent dosage, sorbate initial concentration, and particle size [18]. The influence of pH and ionic strength on mercury elimination was evaluated, for example, in the work of Carro et al. using dry bracken ferns [19]; the effects of pH, initial metal concentration, biosorbent mass, and contact time on mercury removal were investigated by Boutsika et al. using biochar produced from malt spent rootlets [20]; the impacts of initial metal concentration, pH, and competitive ions on the sorption of different metals by alkali-treated rice husks as biosorbents were reported in the work of Krishnani et al. [21]. Usually, sorption experiments are developed in such a way that only one variable or factor is evaluated each time while the others remain constant [22,23,24]. Interactions among target factors are hence poorly explored. Multivariate statistics methods allow reducing experimental efforts and provide information about the impact of individual or combined variables on the obtained responses. In line with this, the design of experiments (DoE) is a fundamental tool for searching the best solution and improving the process efficiency [25]. Response surface methodology (RSM) is a set of techniques that describes the relations between several independent variables and the respective responses. This procedure describes the process and improves its efficacy while reducing costs and experimental time [25,26].

The aim of this study is to use raw *E. globulus* barks to remove mercury from contaminated waters. The specific objectives are as follows: (i) optimizing the conditions of pH, biosorbent dosage, and salinity in the sorption processes; (ii) using the RSM to obtain the appropriate response functions; (iii) adjusting pseudo-first-order (PFO), pseudo-second-order (PSO), and Elovich models for the experimental data in order to obtain information about the applicability of this process for a mercury water treatment proposal.

## 2. Results

### 2.1. Sorbent Characterization

**Figure 1:**
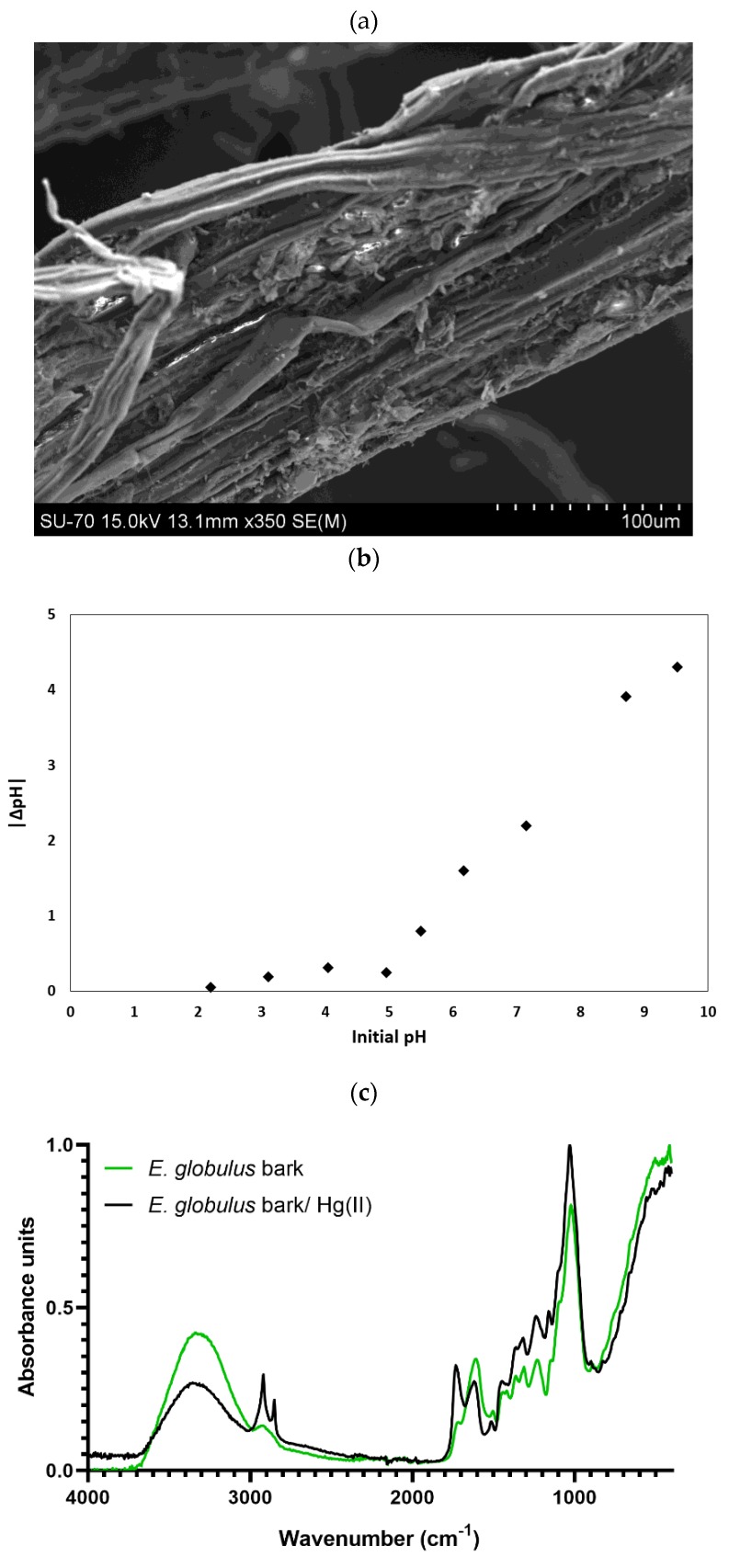
Characterization of the *Eucalyptus globulus* (*E. globulus*) bark utilized in the Hg(II) removal experiments: (**a**) SEM image; (**b**) relationship between |ΔpH| and initial pH; (**c**) FTIR spectra before and after the sorption assays.

The morphology of the biomass of *E. globulus* bark was studied by Scanning Electron Microscopy (SEM), being possible to observe that it was composed of rough fibres with *ca.* 170 µm thickness; pieces of *ca.* 1 cm length were utilized (see Figure 1a; more details are provided in Section 4.2).

The charge of a sorbent surface is another kind of relevant information for sorption processes and is influenced by the pH of contaminated water in contact with biosorbents. The point of zero charge (PZC) was determined, and the plot shown in Figure 1b exhibits the relationship between |ΔpH| and initial pH. According to the method used [27], the PZC appears when ΔpH ≈ 0, at pH 2.2, at which the surface is neutral and the functional groups do not contribute to the pH of the solution. Above the pH value of 2.2, the surface charge becomes negative, and below this value, the sorbent is positively charged [28].

The FTIR spectra of both the *E. globulus* bark prior and after its use in sorption experiments are shown in Figure 1c. This is a nondestructive technique that allows identifying the main functional groups present on the surface of biomass. The appearance, the disappearance, or the displacement of the vibration frequencies after sorption may indicate bonds between the functional groups and the sorbate [15]. The peaks observed at 3300 cm^−1^ are characteristics of –OH and –NH_2_ groups [29]. The emergence of a double peak between 2850 and 2920 cm^−1^ in the loaded biosorbent spectrum is representative of the stretching vibrations of asymmetric and symmetric C–H groups [17], and it suggests their participation in the bonds established between the biosorbent and the Hg(II) in the solution. The band at 1730 cm^−1^ is due to C=O bonds, and the band at 1620 cm^−1^ is attributed to C=C stretching frequencies, which is ascribed, in general, to the vibration of an aromatic ring present in lignin [30]. The other remarkable peaks are represented by the N–H bond of the amino group at 1520 cm^−1^, the C–O–C vibration of the cellulose (bands around 850 cm^−1^) [31] and the C–O stretching of a primary alcohol (peak at 1025 cm^−1^) [29]. The appearance of the peak at 1120 cm^−1^ after the exposure to the Hg(II) may be attributed to the C–O (COOH) vibration [15].

### 2.2. Optimization of the Hg(II) Removal Conditions

**Figure 2:**
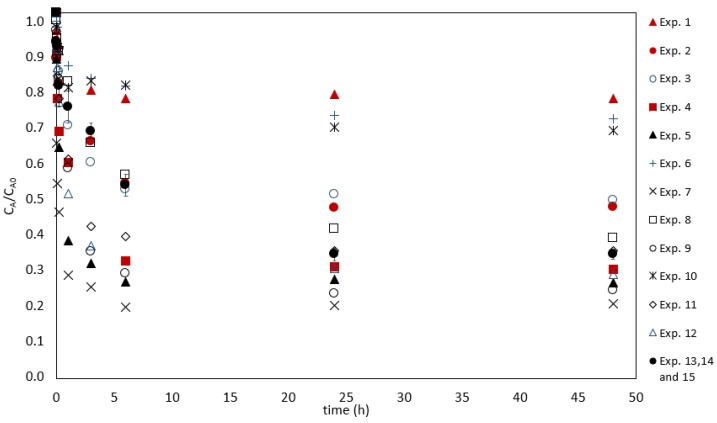
Normalized Hg(II) concentration in a solution as a function of time for different experiments performed according to the conditions (initial Hg(II) concentration of 50 µg dm^−3^, stirring speed of 650 rpm, and a temperature of 22 ± 1 °C).

Figure 2 shows the curves of the experiments performed by a Box–Behnken design. The last three experiments represent replications of the central point, and their averages are shown together with the error bars. The control results are not shown, but the concentrations remained constant, when the times had variation coefficients lower than 10%. The results indicated that the major content of Hg(II) was removed during the first hours followed by a period, where the sorption kinetics was slower towards equilibrium. The driving force promoted by a large mercury concentration gradient between the solution and the biosorbent was higher at the beginning of the process, when all the sorption sites were available [29]. As the process occurred, the sites became occupied, and the sorption tended to reach the equilibrium. Although the normalized final mercury concentration displayed remarkable differences, all the experiments reached equilibrium after six hours.

**Figure 3:**
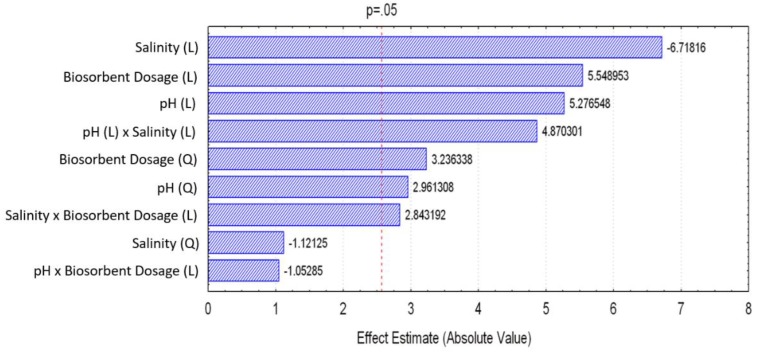
Pareto chart with the impact of the factors studied.

The results of the 15 experiments and the Hg(II) removal percentages are presented in Table 1. The experimental conditions and the codified variables are detailed in Materials and Methods section (see Tables 5 and 6; fixed conditions: temperature of 22 °C, contact time of 48 h, and stirring speed of 650 rpm). Regarding the results of the applied DoE, the minimum response observed (percentage of Hg(II) removal; %) was 23% for experiment 1 (pH 4.0 (level −1), salinity of 15 (level 0), and biosorbent dosage of 0.2 g dm^−3^ (level −1)), and the maximum response was 77% for experiment 7 (pH 6.5 (level 0), salinity of 0 (level −1), and biosorbent dosage of 0.8 g dm^−3^ (level +1)). These values are explained in Figure 3, which presents a pareto chart with the linear (L), quadratic (Q), and interaction effects of the factors, obtained at a 95% confidence level. The significant variables are the ones with score values above the red line (*p*-value ≤ 0.05). The salinity is the most impactful factor, and it contributed negatively to the removal efficiency. The other variables affected positively the results. The linear effect of the variables, the quadratic effect of the pH, and the biosorbent dosage, as well as the salinity–pH interaction and the salinity–biosorbent dosage interaction, were considered significant for the model that describes Hg(II) sorption.

The quadratic effects of the salinity and the interaction between pH and biosorbent dosage were considered nonsignificant (*p*-value > 0.05), and hence, they were eliminated from the full model to obtain the reduced model (RM) only with the impactful factors for the removal efficiency of the sorption. The coefficients obtained for the RM are presented in Table 2. The final uncoded RM (Equation (1)) was obtained by applying Equation (4) (see Section 4.5) for the back-substitution of the variables, and it is presented in Table 3 together with the values of the determination coefficient $R^{2}$ and the adjusted coefficient of determination $R_{\mathrm{Adj}}^{2}$. The value of the determination coefficient, $R^{2}=0.945$, presented for the reduced equation indicates good fit to the experimental data. However, the lower value of the adjusted coefficient of determination, $R_{\mathrm{Adj}}^{2}=0.793$, represents that the goodness of the fit is due to the large number of parameters instead of the robustness of the proposed function.

**Figure 4:**
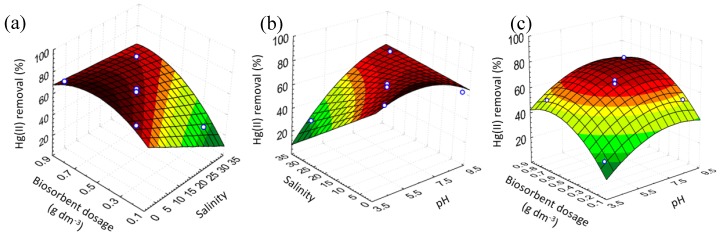
Response surface plots showing the interaction effects of variables on the Hg(II) removal percentage: (**a**) the interaction between biosorbent dosage and salinity; (**b**) the interaction between salinity and pH; (**c**) the interaction between biosorbent dosage and pH. Dots represent experimental values; the Hg(II) removal varies from 0% (dark green) 100% (dark red).

Figure 4 exhibits the 3D response surfaces obtained through the uncoded RM in Table 3 by plotting two variables and remaining constant the value of the other variable at the central point. Figure 4a presents the influence of biosorbent dosage and salinity on the Hg(II) removal percentage, Figure 4b shows the impact of salinity and pH on the Hg(II) removal percentage, and Figure 4c shows the effect of biosorbent dosage and pH on the Hg(II) removal percentage.

It can be observed that, by the sloping profile of the surfaces, the great importance of the concentration of salts in the solution. In addition, more pronounced differences of this variable in the response were observed with lower pH values and biosorbent dosages. The effect of biosorbent dosage variation was more relevant at higher salinity and pH values, and the effect of pH was more impactful at higher salinity and biosorbent dosages. 

### 2.3. Kinetic Modelling

**Figure 5:**
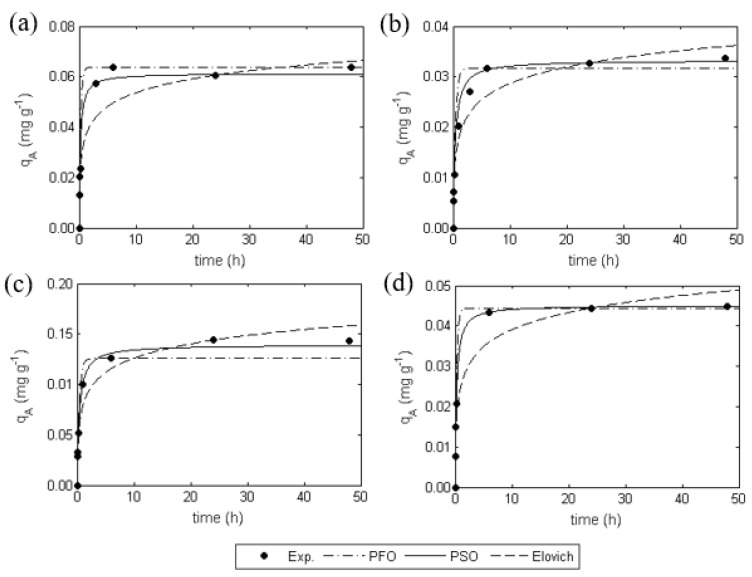
Sorption kinetics modelling on the *E. globulus* bark under different conditions: (**a**) biosorbent dosage of 0.2 g dm^−3^, salinity of 15, and pH of 4.0; (**b**) biosorbent dosage of 0.8 g dm^−3^, salinity of 15, and pH of 4.0; (**c**) biosorbent dosage of 0.2 g dm^−3^, salinity of 15, and pH of 9.0; (**d**) biosorbent dosage of 0.8 g dm^−3^, salinity of 15, and pH of 9.0.

The curves of experiments 1–4 were modelled by applying the most known kinetic models to adjust the results obtained. The fittings are expressed in Figure 5 in terms of the Hg(II) concentration on the biosorbent as a function of time. Table 4 summarizes the calculated values of the different kinetic parameters as well as the coefficients of determination ($R^{2}$) and the average absolute relative deviation (AARD).

Among all the models tested, the PSO equation best describes the experimental results for all the four curves, which suggests that Hg(II) sorption by *E. globulus* bark occurs through a chemical reaction with second-order kinetics. This also can be observed in the high values of $R^{2}$ found (between 0.973 and 0.996) and the low AARDs (3.42% to 9.69%) in Table 4. The parameter values of $q_{Ae}$ calculated from the PSO equation agree well with the observed ones, which is confirmed by the relative errors between them not greater than 3.77%.

Although a slight difference was observed at pH 4, the rate constant $k_{2}$ follows the biosorbent dosage tendency using the PSO model, i.e., higher $k_{2}$ for higher masses of *E. globulus* bark and lower $k_{2}$ for lower masses of *E. globulus* bark. The increase in the biosorbent dosage enhances the number of the available active sorption sites and leads to faster removal of Hg(II) from the solution. The same behavior does not take place using the other models, probably because of the poorer fittings achieved with those models.

## 3. Discussion

The DoE by the application of the Box–Behnken method allowed obtaining relevant outcomes about the influence of different factors on the Hg^2+^ sorption efficiency. The most impactful factor, salinity, has been studied in a range from no salinity to that close to seawater salinity. This wide interval provides information about the behavior of the system from simple to complex matrices, where competitive elements may interfere in the removal process. Indeed, Carro et al. [19] reported a drastic drop of mercury uptake (*ca.* 85%) by bracken ferns in the presence of 58.4 g dm^−3^ of NaCl. In another study using *Cystoseira baccat*, NaCl concentrations of 5.8 and 58.4 g dm^−3^ decreased mercury sorption by 8% and 80%, respectively, for solutions with an initial Hg^2+^ concentration of 500 mg dm^−3^ and pH 6 [32]. In the case of our work, the impact of salinity was also negative, showing the best removal accomplished in experiment 7 (no salinity), in accordance with the abovementioned assays, i.e., ionic competition penalizes Hg(II) sorption.

Salinity not only affects sorption capacity, but also has effects on mercury speciation. In line with previous investigations [6,19], Hg^2+^ ions exhibit high affinity to Cl^−^ ions and tend to form chloro complexes with high stability constants [14]. This phenomenon may be explained by the fact that Hg^2+^ (as a soft cation) coordinates preferentially with soft bases containing chloride as donors, establishing more stable bonds than those between Cl^−^ and hard cations mostly present in natural waters, such as, Na^+^, Mg^2+^, or Ca^2+^ [14].

**Figure 6:**
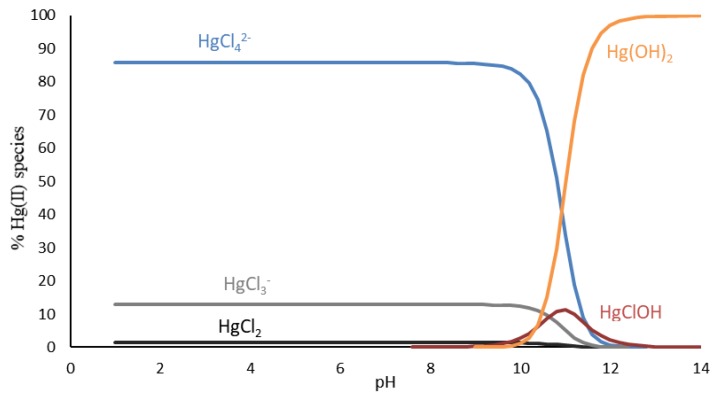
Speciation diagram for Hg(II) in aqueous solutions, with NaCl concentration of 30 g dm^−3^ at a temperature of 22 °C.

Figure 6 presents the speciation of Hg in the solutions containing 30 g dm^−3^ of NaCl as an example of this circumstance. In the NaCl solutions with the pH range shown in this work, Hg was found as neutral and negatively charged complexes, considerably stable, which were more difficult to remove from the solution. Similar to the phenomenon observed in Figure 4a for small biosorbent dosages, the efficiency of sorption reduced significantly with the increase of the salt in the solutions. The effect of the salinity was not impactful, when larger amounts of biosorbent were used, probably due to the higher presence of functional groups on the biosorbent surface interacting with the complexes in the solutions, shifting the equilibrium of the species and contributing to the removal of mercury through the formation of coordinate covalent bonds [14,33].

The same behavior was observed in Figure 4b, the salinity was impactful only at low pH. Acidic media may interfere with the stability of the chloro complexes of mercury in the solutions, and the excess of H^+^ ions possibly interacts with these complexes and consequently impairs their removal from the solutions. Moreover, the ionic state of the functional groups on the *E. globulus* bark surface is mainly affected by pH changes and plays an important role in metal removal [19]. It is important to mention that, in these complexes matrices, several equilibriums are involved in the sorption processes and each case needs to be evaluated separately.

Lastly, the plot of the interaction impact of biosorbent mass and pH is shown in the Figure 4c. The increase in the biosorbent dosage contributed positively to the removal efficiency until an optimal condition was reached. This fact may be ascribed to the formation of agglomerates of the biomass, preventing the access to some sorption sites of the Hg(II) in the solutions. Besides, the gradient of the concentration becomes smaller, as the sorption occurs, and at some point, the driving force may be not strong enough to promote the removal.

The calculations exhibited that the optimized operating conditions for the Hg(II) removal by the *E. globulus* bark under the range studied are shown as follows: biosorbent dosage of 0.55 g dm^−3^, no salinity, and pH 6.0. The metal removal expected under these conditions was 81%. Concerning the biomass features, the excellent performance of this biosorbent may be attributed to the high affinity between the Hg(II) and the functional groups on the solid surface like OH, CH_3_, CH_2_, and C–O.

It is possible to extract, from Figure 4 and the model obtained, important information about the Hg(II) uptake by the *E. globulus* bark for several operational conditions. In many cases, the optimized variables are not the more realistic or applicable conditions to treat real wastewaters. Nevertheless, through the large intervals between the variables conditions, most of the possible scenarios are covered. The salinity of 15 g dm^−3^, for instance, is more frequent in the aquatic or industrial environment, and under this condition, it is possible to achieve a removal efficiency of 74% with a pH value of 8.0 and a biosorbent dosage of 0.7 g dm^−3^. The correction of pH is quite simple, and despite the normal pH of the real wastewaters being around 6.5, this variable can easily be adjusted to the optimized values under low operational costs. The model provides information about the behavior of the process (in a range of conditions studied) and avoids spending time with unnecessary experiments. Another advantage attained with the model is the overview of the interaction between the factors, which means most of the times is ignored and may bring unexpected results in the efficiency of the sorption.

The kinetic curves exhibit the large affinity of the *E. globulus* bark to sorb mercury. In terms of industrial application, sorption time or residence time is an important variable to consider, since it is directly related with profitability of the system. With more time, the energy consumption is higher, the working hour becomes longer, and less volume is treated. The high kinetic constants from the PSO model together with the fast equilibrium time, in which after six hours no relevant removal was observed, highlight this biosorbent is beneficial for use in remediation technologies.

## 4. Materials and Methods

### 4.1. Chemicals

The chemicals used in this work were all of analytical grade, purchased from chemical commercial suppliers and used without any purification. A certified standard solution of mercury(II) nitrate (1000 ± 2 mg dm^−3^), sodium hydroxide (≥99%), and nitric acid (65%) were purchased from Merck, and sodium chloride (≥99%) was acquired from Applichem Panreac. The standards solutions for the calibration curves were obtained by diluting the corresponding stock solutions in high-purity water (18 MΩ cm) or a nitric acid solution (2%). All the glassware used in the experiments was acid-washed prior to use for at least 24 h.

### 4.2. Biomass Characterization

**Figure 7:**
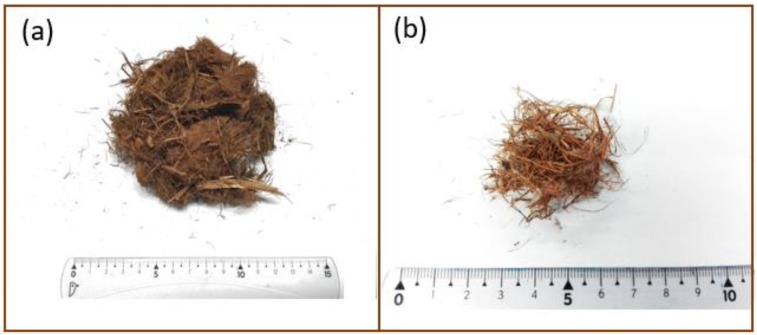
(**a**) *E. globulus* bark provided by The Navigator Company; (**b**) *E. globulus* bark prepared for use in the sorption experiments.

The *E. globulus* bark used in this work was provided by The Navigator Company (Cacia, Portugal), directly from its debarking/crushing unit (see Figure S1, Supplementary Materials). The biosorbent was dried under room temperature and humidity conditions, and it was then cut into pieces with *ca*. 1 cm length (see Figure 7). No additional chemical or thermal pretreatments were applied before the sorption assays. The morphology was assessed by SEM using a Hitachi SU-70 SEM microscope with a Bruker Quantax 400 detector operating at 20 kV. The FTIR spectra of the biosorbent before and after sorption were recorded with a Bruker Tensor 27 spectrometer coupled to a horizontal attenuated total reflectance (ATR) cell using 256 scans at a resolution of 4 cm^−1^. The samples were examined directly, and data were obtained as absorbance from a wavenumber range from 400 to 4000 cm^−1^. The biosorbent PZC was determined according to the immersion method proposed by Fiol and Villaescusa [27] using an incubator shaker HWY-200D.

### 4.3. Chemical Quantification

The pH was recorded on a WTW series 720 m, and the salinity was measured by an Eclipse handheld refractometer model (part number: 45–63).

The mercury quantification was performed by a cold-vapor atomic fluorescence spectroscopy (CV-AFS) on a PSA cold vapour generator (model 10.003), using a Merlin PSA detector (model 10.023) and SnCl_2_ as a reducing agent. In this method, the mercury(II) concentration was obtained as a signal and converted to the desired concentrations through a calibration curve constructed using five standards solutions (0.0, 0.1, 0.2, 0.3, and 0.5 µg of Hg(II) dm^−3^). The calibration curves were plotted at least three times a day, and the obtained determination coefficient was always ≥0.995. Each sample was measured in triplicate with admissible variation coefficients between replicas lower than 10%. The limit of quantification of this technique was 0.02 µg dm^−3^.

### 4.4. Biosorption Experiments

Experiments were performed in batch conditions in 1 dm^−3^ volumetric flasks, magnetically stirred at 650 rpm, under a temperature of 22 ± 1 °C. The capability of the biosorbent to remove Hg(II) was assessed by contacting the biomass with a Hg(II) solution for 48 h (see experimental conditions in Table 5). The mass of *E. globulus* varied between 0.2 and 0.8 g (doses of 0.2–0.8 g dm^−3^), and the initial metal concentration was fixed at 50 µg dm^−3^. These solutions were prepared by diluting the mercury stock solution in tap water to the desired mercury concentration, which is the concentration limit for wastewaters discharges [34]. The salinity was adjusted using NaCl (0–30 g dm^−3^), and pH was fixed between 4 and 9 with NaOH (0.1 mol dm^−3^) or HNO_3_ (0.5 mol dm^−3^). The starting point of the experiments was the time, when the mass of the biosorbent was added into the flasks, and the samples were collected at different times, filtered with a 0.45 µm Millipore filter, adjusted to a pH value of <2 with HNO_3_ and then analyzed for the Hg(II) concentrations in the solutions. A control experiment (without the biosorbent) was always run to check if Hg(II) was sorbed on the vessel walls or lost by volatilization. Experiments 13–15 shown in Table 1 and Table 5 are three replicates carried out to assess the experimental reproducibility and error, being also necessary for the Box–Behnken design.

The average amount of the sorbed Hg(II) per unit mass of solid, $q_{A}$ (mg g^−1^), was calculated by a global material balance at time t in a solution:

(2) $$q_{A}=\frac{\left(C_{A0}-C_{A}\right)V_{L}}{m_{S}}$$

where A denotes Hg(II), $V_{L}$ is the solution volume (dm^3^), $m_{S}$ is the mass of biosorbents (g), $C_{A0}$ (mg dm^−3^) is the initial concentration of Hg(II) in the solution, and $C_{A}$ (mg dm^−3^) is the concentration of Hg(II) at time t.

The removal efficiency was calculated as follows:

(3) $$\mathrm{Removal}\;\left(\%\right)=100\frac{\left(C_{A0}-C_{\mathrm{Af}}\right)}{C_{A0}}$$

where $C_{\mathrm{Af}}$ (mg dm^−3^) is the Hg(II) concentration at the end of the experiment.

### 4.5. Response Surface Methodology

RSM is a statistical tool that describes the relation between several independent factors and one or more responses. The RSM is based on the fit of diverse models (linear functions, quadratic polynomial functions, and others) to the experimental results generated from the DoE and the verification of the model obtained by means of statistical techniques. The aim of the DoE is to improve the efficiency of the process while minimizing the number of experiments without losing the reliability of the results obtained. It reduces the experimental time and consequently the costs involved [25,26].

A Box–Behnken design of 3 factors and 3 levels was selected to evaluate the performance of the *E. globulus* bark as a biosorbent to remove mercury from waters in sorption processes. The factors studied were pH (4, 6.5, and 9), sorbent dosage (0.2, 0.5, and 0.8 g dm^−3^), and salinity (0, 15, and 30), and the response variable was the Hg(II) removal efficiency (%). The performed experimental conditions are listed in Table 5.

The RSM model was operated by using the variables codified to have a common comparison basis. The coded input values are +1, 0, and −1, and they were obtained by transforming the experimental factors using Equation (4) (see Table 6):

(4) $$X_{k}{}_{\;}=\frac{x_{k}{-x}_{0}}{\Delta_{x_{k}}}$$

where $X_{k}$ is the codified value of the independent variable $x_{k}$, $x_{0}$ is the variable value at its center point, and $\Delta_{x_{k}}$ is the step change between levels for the k variable.

The RSM results were obtained by using a second-order polynomial equation written as:

(5) $$Y=\beta_{0}+\sum_{i=1}^{k}\beta_{i}X_{i}+\sum_{i=1}^{k}\beta_{\mathrm{ii}}X_{i}^{2}+\sum_{i<j}^{k}\beta_{\mathrm{ij}}X_{i}X_{j}$$

where Y refers to the response variable studied, $\beta_{0}$ is a constant, $\beta_{i}$, $\beta_{\mathrm{ii}}$, and $\beta_{\mathrm{ij}}$ are the model coefficients associated with linear effects, quadratic effects, and interaction effects, respectively.

The software STATISTICA (version 5.1, StatSoft Inc., Tulsa, OK, USA) was applied to analyze the results. Analysis of variance (ANOVA) was used to assess the significant factors and the interactions with the Fisher’s test and its associated probability p(F), while t-tests were performed to evaluate the significance of the fitted coefficients of each model. The coefficient of determination, $R^{2}$, and the adjusted coefficient of determination, $R_{\mathrm{Adj}}^{2}$, were used to verify goodness of the fit, and they were expressed as follows:

(6) $$R^{2}=1-\frac{\sum {\left(\hat{y_{i}}-y_{i}\right)}^{2}}{\sum {\left(y_{i}-\bar{y}\right)}^{2}}$$

(7) $$R_{\mathrm{adj}}^{2}=1-\left(1-R^{2}\right)\frac{\left(N_{\mathrm{DP}}-1\right)}{\left(N_{\mathrm{DP}}-N_{P}-1\right)}$$

where $N_{\mathrm{DP}}$ is the number of the experimental data, $N_{P}$ is the parameters number, $y_{i}$ is the experimental value, $\hat{y_{i}}$ is the value calculated by the model, and $\bar{y}$ is the mean of the experimental values.

### 4.6. Kinetics Modelling

In order to obtain information about the kinetics of the sorption of Hg(II) onto the biosorbents, three widely used reaction-based models were fitted to the experimental data, namely PFO, PSO, and Elovich models.

The PFO equation was suggested by Lagergren [35] and describes sorption processes as proportional to the distance from the equilibrium $\left(q_{Ae}-q_{A}\right)$, which was shown as follows:

(8) $$\frac{dq_{A}}{dt}=k_{1}\left(q_{Ae}-q_{A}\right),$$

where $k_{1}$ (h^−1^) is the rate constant of the model and $q_{\mathrm{Ae}}$ (mg g^−1^) is the Hg(II) concentration on the solid at equilibrium. After integration from the initial Hg(II) free particle condition (t=0, $q_{A}=0$) to the equilibrium condition ($q_{A}=q_{Ae}$), Equation (7) was given as:

(9) $$\ln\left(q_{Ae}-q_{A}\right)=\;\ln q_{Ae}-k_{1}t$$

The PSO model was represented as [36]:

(10) $$\frac{dq_{A}}{dt}=k_{2}{\left(q_{\mathrm{Ae}}-q_{A}\right)}^{2}$$

where $k_{2}$ (g mg^−1^ h^−1^) is the rate constant of the model. After integration, the Equation (10) was rewritten as:

(11) $$\frac{t}{\;q_{A}}=\frac{1}{k_{2}q_{\mathrm{Ae}}^{2}}+\frac{1}{q_{\mathrm{Ae}}}t$$

The Elovich equation is one of the most useful models to describe reactions involving sorption on heterogeneous surfaces [37], and it is mathematically expressed as:

(12) $$\frac{dq_{A}}{dt}=\alpha e^{-\beta q_{A}}$$

where α is the initial Hg(II) sorption rate (mg g^−1^ h^−1^ and β (g mg^−1^) is the desorption constant of the model. Assuming αβt >>1 and applying the conditions (t=0 to t=t and $q_{A}=0$ to $q_{A}=q_{A}$), Equation (13) was obtained as:

(13) $$q_{A}=\frac{1}{\beta }\ln\left(\alpha \beta \right)+\frac{1}{\beta }\ln t$$

All the parameters of the kinetics models were obtained by nonlinear regression using Matlab R2014a program, and they were optimized by the Nelder–Mead simplex algorithm to minimize the error between the experimental and predicted data. The fittings of the kinetic equations were examined with the coefficient of determination ($R^{2}$) (Equation (6)) and the AARD, which was mathematically expressed by:

(14) $$AARD\left(\%\right)=\frac{100}{N_{\mathrm{DP}}}\sum_{i=1}^{N_{\mathrm{DP}}}\frac{\left|\hat{y_{i}}-y_{i}\right|}{y_{i}}$$

## 5. Conclusions

The impacts of diverse operational conditions on the sorption of Hg(II) from aqueous solutions using *E. globulus* bark as a biosorbent were evaluated. The optimal conditions for metal removal are a pH value of 6.0, no salinity, and a biosorbent dosage of 0.55 g dm^−3^, resulting in 81% of Hg(II) elimination. This remarkable performance noticed by *E. globulus* bark is highly influenced by its chemical characteristics as well as the sorption conditions. Salinity is the most significant factor for the sorption of mercury, and the increase of ionic strength resulted in metal removal reduction. The PSO model is the most appropriate equation to represent the experimental behavior, and the kinetic constants increased with the increasing biosorbent dosage.

Taking into account the environmental concerns, the use of small masses of a sorbent promotes less generation of contaminated wastes and affords more sustainable and eco-friendly systems. The use of very small masses in this work evidenced the great affinity of *E. globulus* bark to Hg(II). The innovative application of the RSM model to describe more realistic conditions provides an insight into an effective implementation of this biosorbent in wastewater cleanup technologies.

## Figures and Tables

**Table 1:** Results of the experiments performed according to the Box–Behnken design, along with noncodified (Table 5) and codified (Table 6) conditions. Fixed conditions: temperature of 22 °C, contact time of 48 h, and stirring speed of 650 rpm.

Experiment	pH	Salinity	Biosorbent Dosage (g dm^−3^)/(-)	Removal (%)
1	4.0 (−1)	15 (0)	0.2 (−1)	23
2	9.0 (+1)	15 (0)	0.2 (−1)	53
3	4.0 (−1)	15 (0)	0.8 (+1)	51
4	9.0 (+1)	15 (0)	0.8 (+1)	70
5	6.5 (0)	0 (−1)	0.2 (−1)	74
6	6.5 (0)	30 (+1)	0.2 (−1)	29
7	6.5 (0)	0 (−1)	0.8 (+1)	77
8	6.5 (0)	30 (+1)	0.8 (+1)	62
9	4.0 (−1)	0 (−1)	0.5 (0)	76
10	4.0 (−1)	30 (+1)	0.5 (0)	32
11	9.0 (+1)	0 (−1)	0.5 (0)	65
12	9.0 (+1)	30 (+1)	0.5 (0)	71
13	6.5 (0)	15 (0)	0.5 (0)	68
14	6.5 (0)	15 (0)	0.5 (0)	65
15	6.5 (0)	15 (0)	0.5 (0)	65

**Table 2:** Regressed coefficients of Equation (5) (see Section 4.5) obtained for the reduced model and the individual significance.

Coefficients of Equation (5)	Reduced Model	*p*-Value
$\beta_{0}$	67.9	2.63 × 10^−8^
$\beta_{1}$	9.6	1.33 × 10^−3^
$\beta_{2}$	−12.2	3.20 × 10^−4^
$\beta_{3}$	10.1	1.00 × 10^−3^
$\beta_{11}$	−8.2	2.05 × 10^−2^
$\beta_{33}$	−8.9	1.41 × 10^−2^
$\beta_{12}$	12.5	2.09 × 10^−3^
$\beta_{23}$	7.3	2.76 × 10^−2^

**Table 3:** Response with the uncoded reduced model for a certain coefficient of determination and a certain adjusted coefficient of determination.

Response with the Uncoded Reduced Model	$R^{2}$	$R_{Adj}^{2}$	Equation
$Removal\;\left(\%\right)=3.1+15.8pH-3.8Salinity+108.0Biosorbent\;dosage-1.3pH^{2}-99.0Biosorbent\;dosage^{2}+0.3\left(pH\times Salinity\right)+1.6\left(Salinity\times Biosorbent\;dosage\right)$	0.954	0.793	(1)

**Table 4:** Kinetic fitting parameters for the Hg(II) sorption.

	Pseudo-First-Order (PSO)				Pseudo-Second-Order (PSO)				Elovich			
**pH 4.0**	$k_{1}$ **(h^−1^)**	$q_{e}$ **(mg g^−1^)**	$R^{2}$	AARD **(%)**	$k_{2}$ **(g mg^−1^ h^−1^)**	$q_{e}$ **(mg g^−1^)**	$R^{2}$	AARD **(%)**	**α (mg g^−1^ h^−1^)**	β **(g mg^−1^)**	$R^{2}$	AARD **(%)**
0.2 g dm^−3^	3.81	0.0652	0.939	12.1	80.0	0.0613	0.973	7.73	0.667	126.0	0.918	8.10
0.8 g dm^−3^	2.52	0.0317	0.908	15.8	81.3	0.0333	0.975	9.69	0.200	211	0.957	8.01
**pH 9.0**	$k_{1}$ **(h^−1^)**	$q_{e}$ **(mg g^−1^)**	$R^{2}$	AARD **(%)**	$k_{2}$ **(g mg^−1^ h^−1^)**	$q_{e}$ **(mg g^−1^)**	$R^{2}$	AARD **(%)**	**α (mg g^−1^ h^−1^)**	β **(g mg^−1^)**	$R^{2}$	AARD **(%)**
0.2 g dm^−3^	2.57	0.1260	0.945	13.7	18.5	0.1390	0.983	8.69	0.927	48.5	0.959	11.20
0.8 g dm^−3^	3.78	0.0444	0.979	5.9	90.5	0.0452	0.996	3.42	0.355	163.0	0.955	10.70

**Table 5:** Experimental conditions of the Box–Behnken design. Fixed conditions: temperature of 22 °C, contact time of 48 h, stirring velocity of 650 rpm, and volume of 1 dm^3^.

Experiment	pH	Salinity	Biosorbent Mass (g) or Dosage (g dm^−3^)
1	4.0	15	0.2
2	9.0	15	0.2
3	4.0	15	0.8
4	9.0	15	0.8
5	6.5	0	0.2
6	6.5	30	0.2
7	6.5	0	0.8
8	6.5	30	0.8
9	4.0	0	0.5
10	4.0	30	0.5
11	9.0	0	0.5
12	9.0	30	0.5
13	6.5	15	0.5
14	6.5	15	0.5
15	6.5	15	0.5

**Table 6:** Three different levels and their corresponding experimental conditions for the three different factors studied.

Variable	Level		
	−1	0	+1
pH	4.0	6.5	9.0
Salinity	0	15	30
Biosorbent dosage (g dm^−3^)	0.2	0.5	0.8

## Supplementary Materials

Supplementary materials can be found at https://www.mdpi.com/1422-0067/20/23/5973/s1. Figure S1: Pictures of the debarking unit of *Eucalyptus globulus* trees of The Navigator Company (Cacia, Portugal).

## References

[1] Substance Priority List|ATSDR. https://www.atsdr.cdc.gov/spl/.

[2] (2013). Directive 2013/39/EU of the European Parliament and of the Council of 12 August 2013 amending Directives 2000/60/EC and 2008/105/EC as regards priority substances in the field of water policy. Off. J. Eur. Union.

[3] Baeyens W., Ebinghaus R., Vasiliev O. (1996). Global and Regional Mercury Cycles: Sources, Fluxes and Mass Balances.

[4] Romera E., González F., Ballester A., Blázquez M.L., Muñoz J.A. (2007). Comparative study of biosorption of heavy metals using different types of algae. Bioresour. Technol..

[5] Lopes C.B., Lito P.F., Cardoso S.P., Pereira E., Duarte A.C., Silva C.M., Inamuddin, Luqman M. (2012). Metal recovery, separation and/or pre-concentration. Ion Exchange Technology II—Applications.

[6] Lopes C.B., Otero M., Coimbra J., Pereira E., Rocha J., Lin Z., Duarte A. (2007). Removal of low concentration Hg2+ from natural waters by microporous and layered titanosilicates. Microporous Mesoporous Mater..

[7] Panayotova M.I. (2001). Kinetics and thermodynamics of copper ions removal from wastewater by use of zeolite. Waste Manag..

[8] Nguyen T.A.H., Ngo H.H., Guo W.S., Zhang J., Liang S., Yue Q.Y., Li Q., Nguyen T.V. (2013). Applicability of agricultural waste and by-products for adsorptive removal of heavy metals from wastewater. Bioresour. Technol..

[9] Jiménez-Cedillo M.J., Olguín M.T., Fall C., Colin-Cruz A. (2013). As(III) and As(V) sorption on iron-modified non-pyrolyzed and pyrolyzed biomass from Petroselinum crispum (parsley). J. Environ. Manag..

[10] Zafar M.N., Nadeem R., Hanif M.A. (2007). Biosorption of nickel from protonated rice bran. J. Hazard. Mater..

[11] Balderas-Hernández P., Roa-Morales G., Ramírez-Silva M.T., Romero-Romo M., Rodríguez-Sevilla E., Esparza-Schulz J.M., Juárez-Gómez J. (2017). Effective mercury(II) bioremoval from aqueous solution, and its electrochemical determination. Chemosphere.

[12] Aman A., Ahmed D., Asad N., Masih R., Muhammad Abd ur Rahman H. (2018). Rose biomass as a potential biosorbent to remove chromium, mercury and zinc from contaminated waters Rose biomass as a potential biosorbent to remove chromium, mercury and zinc from contaminated waters. Int. J. Environ. Stud..

[13] Soto-Ríos P.C., León-Romero M.A., Sukhbaatar O., Nishimura O. (2018). Biosorption of Mercury by Reed (Phragmites australis) as a Potential Clean Water Technology. Water Air Soil Pollut..

[14] Stumm W., Morgan J.J., Schnoor J.L., Zehnder A. (1996). Aquatic Chemistry: Chemical Equilibria and Rates in Natural Waters.

[15] Dwivedi A.D., Dubey S.P., Gopal K., Sillanpää M. (2011). Strengthening adsorptive amelioration: Isotherm modeling in liquid phase surface complexation of Pb (II) and Cd (II) ions. Desalination.

[16] Patnukao P., Kongsuwan A., Pavasant P. (2008). Batch studies of adsorption of copper and lead on activated carbon from Eucalyptus camaldulensis Dehn. bark. J. Environ. Sci..

[17] Choudhary B., Paul D., Singh A., Gupta T. (2017). Removal of hexavalent chromium upon interaction with biochar under acidic conditions: Mechanistic insights and application. Environ. Sci. Pollut. Res..

[18] Neris J.B., Luzardo F.H.M., da Silva E.G.P., Velasco F.G. (2019). Evaluation of adsorption processes of metal ions in multi-element aqueous systems by lignocellulosic adsorbents applying different isotherms: A critical review. Chem. Eng. J..

[19] Carro L., Anagnostopoulos V., Lodeiro P., Barriada J.L., Herrero R., Sastre de Vicente M.E. (2010). A dynamic proof of mercury elimination from solution through a combined sorption–reduction process. Bioresour. Technol..

[20] Boutsika L.G., Karapanagioti H.K., Manariotis I.D. (2014). Aqueous mercury sorption by biochar from malt spent rootlets. Water. Air Soil Pollut..

[21] Krishnani K.K., Meng X., Christodoulatos C., Boddu V.M. (2008). Biosorption mechanism of nine different heavy metals onto biomatrix from rice husk. J. Hazard. Mater..

[22] Devani M.A., Munshi B., Oubagaranadin J.U.K. (2015). Characterization and use of chemically activated Butea monosperma leaf dust for mercury(II) removal from solutions. J. Environ. Chem. Eng..

[23] Eom Y., Won J.H., Ryu J.-Y., Lee T.G. (2011). Biosorption of mercury(II) ions from aqueous solution by garlic (Allium sativum L.) powder. Korean J. Chem. Eng..

[24] Vinod V.T.P., Sashidhar R.B., Sivaprasad N., Sarma V.U.M., Satyanarayana N., Kumaresan R., Rao T.N., Raviprasad P. (2011). Bioremediation of mercury (II) from aqueous solution by gum karaya (Sterculia urens): A natural hydrocolloid. Desalination.

[25] Witek-Krowiak A., Chojnacka K., Podstawczyk D., Dawiec A., Pokomeda K. (2014). Application of response surface methodology and artificial neural network methods in modelling and optimization of biosorption process. Bioresour. Technol..

[26] Montgomery D.C. (2001). Design and Analysis of Experiments.

[27] Fiol N., Villaescusa I. (2009). Determination of sorbent point zero charge: Usefulness in sorption studies. Environ. Chem. Lett..

[28] Marcilla A., Beltrán M.I., Navarro R. (2007). Application of TG/FTIR to the study of the regeneration process of husy and HZSM5 zeolites. J. Therm. Anal. Calorim..

[29] Rocha L.S., Almeida Â., Nunes C., Henriques B., Coimbra M.A., Lopes C.B., Silva C.M., Duarte A.C., Pereira E. (2016). Simple and effective chitosan based films for the removal of Hg from waters: Equilibrium, kinetic and ionic competition. Chem. Eng. J..

[30] Liang S., McDonald A.G. (2014). Chemical and thermal characterization of potato peel waste and its fermentation residue as potential resources for biofuel and bioproducts production. J. Agric. Food Chem..

[31] Rafatullah M., Sulaiman O., Hashim R., Ahmad A. (2009). Adsorption of copper (II), chromium (III), nickel (II) and lead (II) ions from aqueous solutions by meranti sawdust. J. Hazard. Mater..

[32] Herrero R., Lodeiro P., Rey-Castro C., Vilariño T., Sastre De Vicente M.E. (2005). Removal of inorganic mercury from aqueous solutions by biomass of the marine macroalga Cystoseira baccata. Water Res..

[33] Atkins P., Jones L. (2007). Chemical Principles.

[34] (2008). Directive 2008/105/EC of the European Parliament and of the Council of 16 December 2008 on environmental quality standards in the field of water policy, amending and subsequently repealing Council Directives 82/176/EEC, 83/513/EEC, 84/156/EEC, 84/491/EEC. Off. J. Eur. Commun..

[35] Lagergren S. (1898). Zur theorie der sogenannten adsorption gel Zur theorie der sogenannten adsorption gelster stoffe, Kungliga Svenska Vetenskapsakademiens. Handlingar.

[36] Ho Y.S., McKay G. (1999). Pseudo-second order model for sorption processes. Process Biochem..

[37] Roginsky S., Zeldovich Y.B. (1934). The catalytic oxidation of carbon monoxide on manganese dioxide. Acta Phys. Chem. USSR.

