# Supplementary material for: Experimental Measurement and Modeling of Hg(II) Removal from Aqueous Solutions Using Eucalyptus globulus Bark: Effect of pH, Salinity and Biosorbent Dosage
Source: Int J Mol Sci. 2019 Nov 27;20(23):5973. doi: 10.3390/ijms20235973 (PMC6929165; doi:10.3390/ijms20235973)
Supplement: Supplementary file 1 [file ijms-20-05973-s001.pdf]

Supplementary Material

of

Experimental measurement and modeling of Hg(II) removal from aqueous solutions using Eucalyptus globulus bark: effect of pH, salinity and biosorbent dosage

Elaine Fabre[a], Carlos Vale[b], Eduarda Pereira[c], Carlos M. Silva[d]*

[a] CICECO, CESAM, University of Aveiro, Aveiro, Portugal (elainefabre@ua.pt);[b] CIIMAR, University of Porto, Matosinhos, Portugal (carlos.vale@ciimar.up.pt);[c] CESAM & LAQV-REQUIMTE, University of Aveiro, Aveiro, Portugal (eduper@ua.pt);[d] CICECO, University of Aveiro, Aveiro, Portugal (carlos.manuel@ua.pt).

* Corresponding author: carlos.manuel@ua.pt

***Eucalyptus globulus* bark for Hg(II) sorption removal**

The biosorbent used in this work for the mercury(II) removal experiments was provided by *The Navigator Company* (Cacia, Portugal) directly from its debarking/crushing unit without any further pretreatments accomplished. Some pictures of the industrial debarking process are shown in Figure S1.

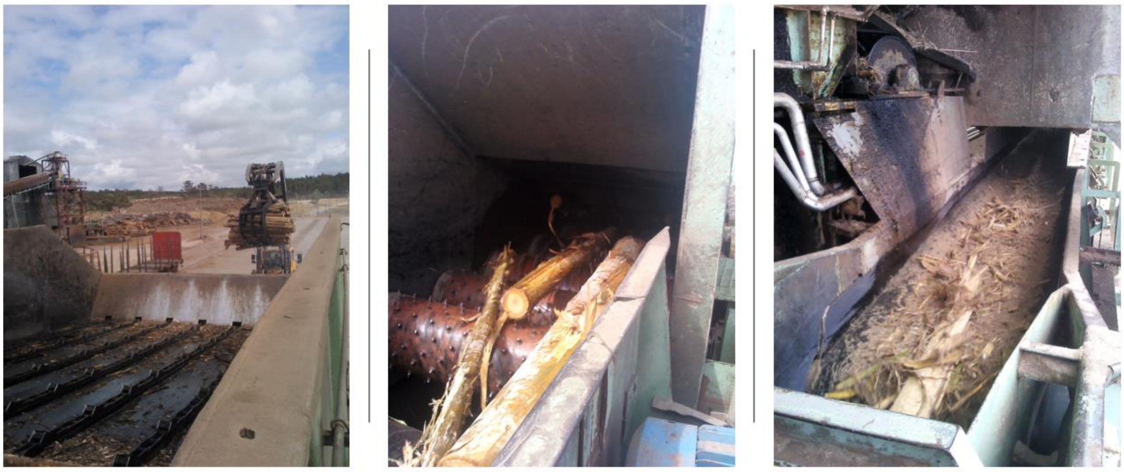

Figure S1. Pictures of the debarking unit of *Eucalyptus globulus* trees of The Navigator Company (Cacia, Portugal).
